# Enhanced Photocatalysis over SrTiO_3_ Nanoparticles Modified with Highly Dispersed Nickel Species

**DOI:** 10.3390/ma19183914

**Published:** 2026-09-15

**Authors:** Yanlong Gao, Hongfei Wu, Senwei Wu, Xinhong Chu, Xiujian Zhao, Gopinathan Sankar, Shouqin Tian

**Affiliations:** 1 State Key Laboratory of Advanced Glass Materials, Wuhan University of Technology, Wuhan 430070, China; 2 College of Intelligent Manufacturing and Materials & Chemical Engineering, Yichun University, Yichun 336000, China; 3 State Key Laboratory of Silicate Materials for Architectures, Wuhan University of Technology, Wuhan 430070, China; 4 Department of Chemistry, University College London, London WC1H 0AJ, UK

**Keywords:** strontium titanate, highly dispersed nickel, photocatalysis, methylene blue degradation

## Abstract

The photocatalytic decomposition of methylene blue (MB) from textile wastewater is promising, though many photocatalysts suffer from a wide band gap, unspecific catalytic active sites and inefficient charge carrier separation. Here, a series of SrTiO_3_-based catalysts modified with highly dispersed nickel species were synthesized via a facile annealing process in a NH_3_ atmosphere obtained from the decomposition of ammonium bicarbonate. The loading amount of nickel is precisely regulated by controlling the concentration of a nickel nitrate hexahydrate solution. The sample containing 0.5 wt% Ni showed a complete degradation of MB under the irradiation of a xenon lamp for 75 min, exhibiting the best performance with an apparent reaction rate constant (k) 3.2 times higher than that of pure SrTiO_3_. The enhancement in photocatalytic performance can probably be attributed to the strong metal–support interaction between the highly dispersed Ni species and SrTiO_3_, which can modulate the band structure by adjusting the concentration of oxygen vacancies, suppress charge recombination, and thus promote efficient carrier separation. Furthermore, the highly dispersed Ni species appears to enhance the adsorption and activation of the reactants on the catalytic surface. This work demonstrates the potential of SrTiO_3_ as a support for a range of highly dispersed catalysts with active metal species that can provide new insights and a practical foundation for the rational design of high-performance SrTiO_3_-based composite photocatalytic materials.

## 1. Introduction

In the 21st century, while the textile industry remains a vital component of human life by fulfilling basic needs, it is also recognized as a primary driver of global environmental contamination [1]. Among various pollutants, methylene blue (MB) dye presents a significant challenge in wastewater treatment due to its water solubility, recalcitrance, and persistence [2,3]. MB not only reduces light penetration in water bodies, harming aquatic ecosystems, but also poses threats to human health through skin irritation, organ damage, and potential carcinogenic risks [4]. Current technologies for MB removal, including adsorption, biodegradation, and membrane separation, are often limited by the dye’s thermal/photo-stability and non-biodegradable nature [5,6]. Semiconductor photocatalysis has garnered considerable attention as an alternative owing to its cost-effectiveness and eco-friendliness [7,8]. This technology utilizes light energy to mineralize MB into harmless small molecules (H_2_O and CO_2_), demonstrating promising application prospects [9].

Perovskite-phase SrTiO_3_ has attracted considerable attention in the field of photocatalysis, primarily due to its chemical inertness, low synthesis cost, structural stability, and photoresponse characteristics in the ultraviolet region, which collectively make it an important material for semiconductor photocatalysts. However, its practical application is constrained by its wide band gap and the rapid recombination of photogenerated charge carriers [10]. Various modification strategies have been explored to enhance the photocatalytic activity of SrTiO_3_, such as the introduction of noble metals or metal oxides [11], elemental doping [12], morphological control [13], and surface modification [14]. The catalytic efficiency and selectivity of metal nanoparticles are currently not ideal, attributed to the fact that only a small portion of the particles possess catalytic functionality while a large number of mismatched particles not only fail to participate in the main reaction but may also promote the formation of by-products [15].

Currently, obtaining a highly dispersed distribution of metal species on supports has become a key strategy to overcome these limitations as this methodology can improve the catalytic efficiency through accessing the active sites efficiently and the preparation of these materials can be cost-effective and it is possible to promote strong metal–support interactions that confer unique electronic properties [16]. This structure not only improves light absorption efficiency and accelerates the separation and transport of photogenerated charges but it also increases the density of free carriers in the vicinity of the Fermi level [17], thereby enhancing photocatalytic activity. In addition, when metallic species are present in a highly dispersed state, their synergistic interaction with the support can effectively promote the adsorption and activation of reactant molecules.

The widespread application of metal oxides in catalytic research is closely linked to their unique functional properties, including abundant reserves, low cost, diverse structures, high tunability, excellent chemical and electrochemical stability, strong metal–support interactions, and facile synthesis [18]. Studies have shown that defect sites (e.g., steps, corners, and vacancies)—especially oxygen vacancies created due to Ni^2+^ interaction with the Ti^4+^ sites—along with surface -OH groups on metal oxides, are capable of serving as anchoring sites for the stabilization of highly dispersed metal entities [19]. A synergistic effect exists between the metal species and the metal oxide support, which profoundly influences the catalytic performance [20]. Previous research [21] demonstrated that defects on metal oxides could act as anchoring sites for highly dispersed iridium species, highlighting how the metal–support interaction modulates the geometric and electronic structure of the central Ir species, thereby affecting its catalytic performance. Zhang et al. [22] prepared a high-performance Ni_0.034_@TiO_2_ catalyst with highly dispersed nickel as the active component and TiO_2_ as the support. The outstanding catalytic activity was ascribed to the cooperative interplay among highly dispersed Ni species, plentiful oxygen vacancies, and multivalent Ti ions, with the role of atomically dispersed nickel being the most significant. Similarly, Wang et al. [23] synthesized a catalyst with highly dispersed iron species via atomic layer deposition (ALD) for the photocatalytic degradation of methylene blue. The results indicated that the iron-modified catalyst significantly enhanced photocatalytic performance by reducing the band gap of TiO_2_ from 3.22 eV to 3.03 eV.

It is noteworthy that although SrTiO_3_ has garnered considerable interest in catalysis due to its tunable band structure, excellent (photo)chemical stability, abundant oxygen vacancies, and inherent catalytic activity, its application as a support for highly dispersed metal species in the photocatalytic degradation of dyes remains a topic that could be further studied. 

The novelty of this work lies in the following aspects: First, we developed a facile and controllable synthesis route, enabling the preparation of a series of SrTiO_3_-based photocatalysts with highly dispersed Ni species. Second, through multi-faceted characterization and mechanistic studies, we elucidated the interaction between the highly dispersed Ni species and the SrTiO_3_ support, which modulates the band structure by increasing the concentration of oxygen vacancies and suppresses charge recombination, thereby promoting efficient carrier separation. Third, our optimized 0.5 wt% Ni/SrTiO_3_ catalyst exhibits excellent activity and stability for methylene blue degradation, outperforming many reported SrTiO_3_-based catalysts. This work not only demonstrates an efficient photocatalyst but also offers fresh perspectives and an experimental basis for the rational construction of high-performance SrTiO_3_-based composite photocatalysts.

## 2. Experiment and Characterization

### 2.1. Reagents

All chemical reagents employed in this work were of analytical purity. Tetrabutyl titanate (C_16_H_36_O_4_Ti, TBOT, 98%), ethylene glycol (C_2_H_6_O_2_, EG), sodium hydroxide (NaOH), absolute ethanol (C_2_H_5_OH), ammonium bicarbonate (NH_4_HCO_3_), isopropanol (IPA), and benzoquinone (BQ) were purchased from China National Pharmaceutical Group Chemical Reagent Co., Ltd. (Shanghai, China). Strontium nitrate (Sr(NO_3_)_2_) and nickel nitrate hexahydrate (Ni(NO_3_)_2_·6H_2_O) were obtained from Aladdin Reagent (Shanghai) Co., Ltd. (Shanghai, China). Deionized water was produced in the laboratory.

### 2.2. Synthesis of Ni/SrTiO_3_ Series Samples

#### 2.2.1. Preparation of SrTiO_3_

Titanium butoxide (TBOT, 2.5 mmol) was added to a beaker containing 60 mL of ethylene glycol (EG) and stirred thoroughly. Meanwhile, 2.5 mmol of strontium nitrate (Sr(NO_3_)_2_) was added to a beaker containing 5 mL of deionized water and mixed thoroughly. Subsequently, the fully dissolved strontium nitrate aqueous solution was slowly added dropwise into the TBOT/EG mixture, followed by the addition of 2.5 mL of a 10 M sodium hydroxide (NaOH) solution. After thorough mixing, the resulting mixture was transferred to a high-pressure autoclave lined with polytetrafluoroethylene and reacted under hydrothermal conditions at 180 °C for 24 h. Finally, the product was collected, washed repeatedly with deionized water and ethanol by centrifugation, and dried at 80 °C to obtain SrTiO_3_ powder.

#### 2.2.2. Preparation of Ni/SrTiO_3_

A certain amount of nickel nitrate hexahydrate (Ni(NO_3_)_2_·6H_2_O) was dissolved in 30 mL of deionized water in a beaker with thorough stirring. The prescribed quantity of as-prepared SrTiO_3_ powder (see Table S1 for the exact amounts used for each target Ni loading) was subsequently introduced into the solution. The resulting suspension was first treated with ultrasound for 30 min, and then vigorously agitated for 5 h. The solid was then separated by centrifugation, washed, and dried. The obtained powder was thoroughly mixed with an excess of ammonium bicarbonate (NH_4_HCO_3_) in a crucible to ensure a sustained generation of a NH_3_ atmosphere during the annealing process, and the crucible was then placed in a tube furnace and the mixture was annealed at 500 °C for 2 h with a heating rate of 5 °C·min^−1^ under vacuum, yielding the final Ni/SrTiO_3_ catalysts. During this process, the in situ generated NH_3_ atmosphere effectively inhibits the aggregation of Ni species and promotes their highly dispersed state, while the mildly reducing atmosphere facilitates the formation of oxygen vacancies on the SrTiO_3_ surface, thereby modulating the electronic structure of the catalyst.

### 2.3. Characterization

X-ray diffraction (XRD) patterns of the samples were obtained using a Bruker D8 Advance diffractometer (Karlsruhe, Germany) with Cu Kα radiation over a 2*θ* range of 20–80°. The morphology of the samples was characterized by high-resolution scanning electron microscopy (HRSEM) on a Zeiss Ultra Plus microscope (Carl Zeiss AG, Oberkochen, Germany). The microstructure and elemental distribution were further analysed using field-emission high-resolution transmission electron microscopy (HRTEM, JEM-2100F, JEOL, Tokyo, Japan) and an energy-dispersive X-ray spectroscopy (EDS) system. Fourier transform infrared (FT-IR) spectra were recorded in the range of 4000–400 cm^−1^ using a Nicolet 6700 spectrometer (Thermo Fisher Scientific, Waltham, MA, USA). Ultraviolet–visible (UV-Vis) diffuse reflectance spectra (DRSs) were collected on a PerkinElmer Lambda 750 S spectrophotometer (PerkinElmer, Shelton, CT, USA). X-ray photoelectron spectroscopy (XPS) measurements were performed on a Thermo Scientific K-Alpha spectrometer (Thermo Scientific, Orlando, FL, USA). Electron paramagnetic resonance (EPR) spectra were acquired using a Bruker EMXplus-6/1 spectrometer (Bruker BioSpin GmbH, Karlsruhe, Germany). Photoluminescence (PL) spectra of the Ni/SrTiO_3_ series samples were obtained using a time-resolved fluorescence spectrometer (QM/TM/NIR).

### 2.4. Photocatalytic Experiments

Photocatalyst (50 mg) was dispersed in 50 mL of a methylene blue solution (10 mg/L) and placed in a quartz glass reactor (inner diameter: 5 cm, height: 6 cm, effective volume: approximately 60 mL). The reactor was wrapped with aluminium foil and placed on a magnetic stirrer. Dark treatment was performed under light-shielded conditions for 45 min to achieve adsorption–desorption equilibrium. After dark treatment, the light source was turned on to start the photocatalytic reaction. A 500 W xenon lamp with an emission spectrum range of 200–2500 nm was used as the simulated sunlight source. The irradiance measured at the centre of the reactor was approximately 150 mW·cm^−2^. The distance between the lamp and the reactor was fixed at 5 cm. During illumination, samples of approximately 3.0 mL of the suspension were taken every 15 min and filtered through a 0.22 μm membrane to remove photocatalyst particles. The concentration of methylene blue in the filtrate was determined at 667 nm using a UV–visible spectrophotometer. In the radical-scavenging experiments, the concentrations of isopropanol (IPA) and benzoquinone (BQ) were both 1 mmol/L.

## 3. Results and Discussion

First, we show the general characterization of the photocatalysts, followed by optical spectroscopic measurements, before we discuss the photocatalytic results.

### 3.1. Structural Characterization of Ni/SrTiO_3_ Series Samples

**Figure 1:**
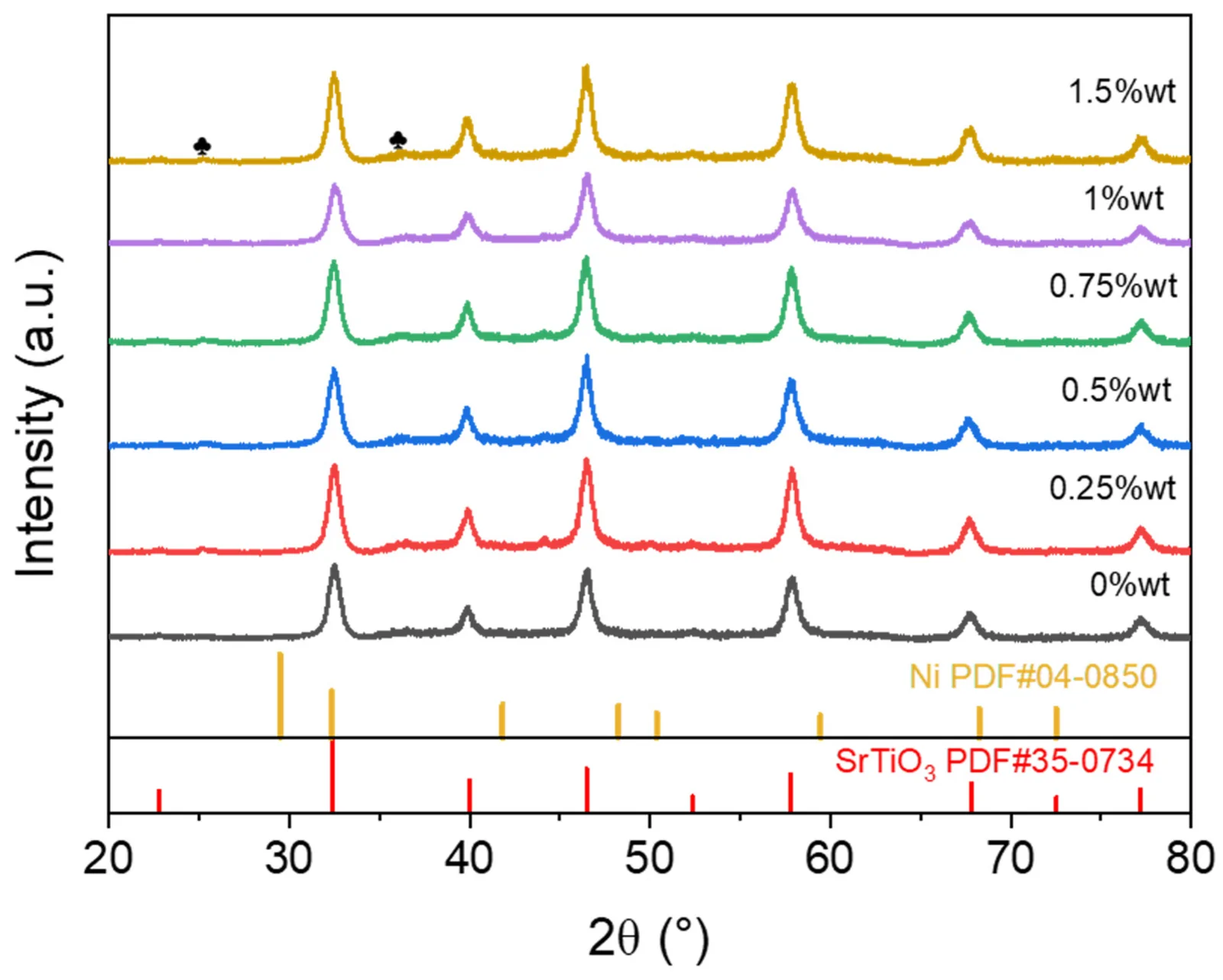
XRD patterns of the Ni/SrTiO_3_ series samples (♣-SrCO_3_ PDF#05-0418).

Figure 1 shows the XRD patterns of the Ni/SrTiO_3_ samples. Diffraction peaks can be observed in all spectra at 32.35°, 39.91°, 46.42°, 57.72°, 67.74°, and 77.09°, which correspond to the (110), (111), (200), (211), (220), and (310) crystal plane reflections of the cubic perovskite SrTiO_3_ (JCPDS 35-0734), respectively [24]. This confirms the successful synthesis of phase-pure cubic SrTiO_3_. The average grain sizes of the samples calculated using the Scherrer formula all fall within the range of 14–17 nm [25]. The weak diffraction peaks observed at 25° and 36° may originate from a small fraction of SrCO_3_ (♣-PDF#05-0418), an impurity that could have formed from the reaction of residual ethylene glycol and Sr species on the surface during the annealing process. The quantity of this impurity phase is almost negligible and does not affect the original crystal structure of SrTiO_3_ (see Figure S1 in Supporting Information for detailed analysis). With increasing Ni loading, no characteristic diffraction peaks corresponding to crystalline Ni or NiO phases are observed, suggesting that the Ni species are likely in a highly dispersed state on the SrTiO_3_ support rather than forming aggregated crystals [25].

The SEM results (Figure S2) indicate that both the pure SrTiO_3_ and the 0.5 wt% Ni/SrTiO_3_ samples possess a short rod-like agglomerated morphology. To further quantify the particle size of the samples, systematic particle size statistical analysis (statistical sample size > 200) was performed based on SEM images for pure SrTiO_3_ and the 0.5 wt% Ni/SrTiO_3_ sample. The particle size distribution histogram (Figure S3 in the Supporting Information) indicates that the samples are primarily distributed within the range of 20–60 nm (the values are larger than the grain sizes obtained from XRD analysis, indicating that the observed particles may consist of multiple aggregated grains). The negligible morphological difference between them implies that Ni loading did not lead to the formation of macroscopic or sub-micron deposits on the carrier surface, nor did it disrupt the inherent structure of the SrTiO_3_ support.

**Figure 2:**
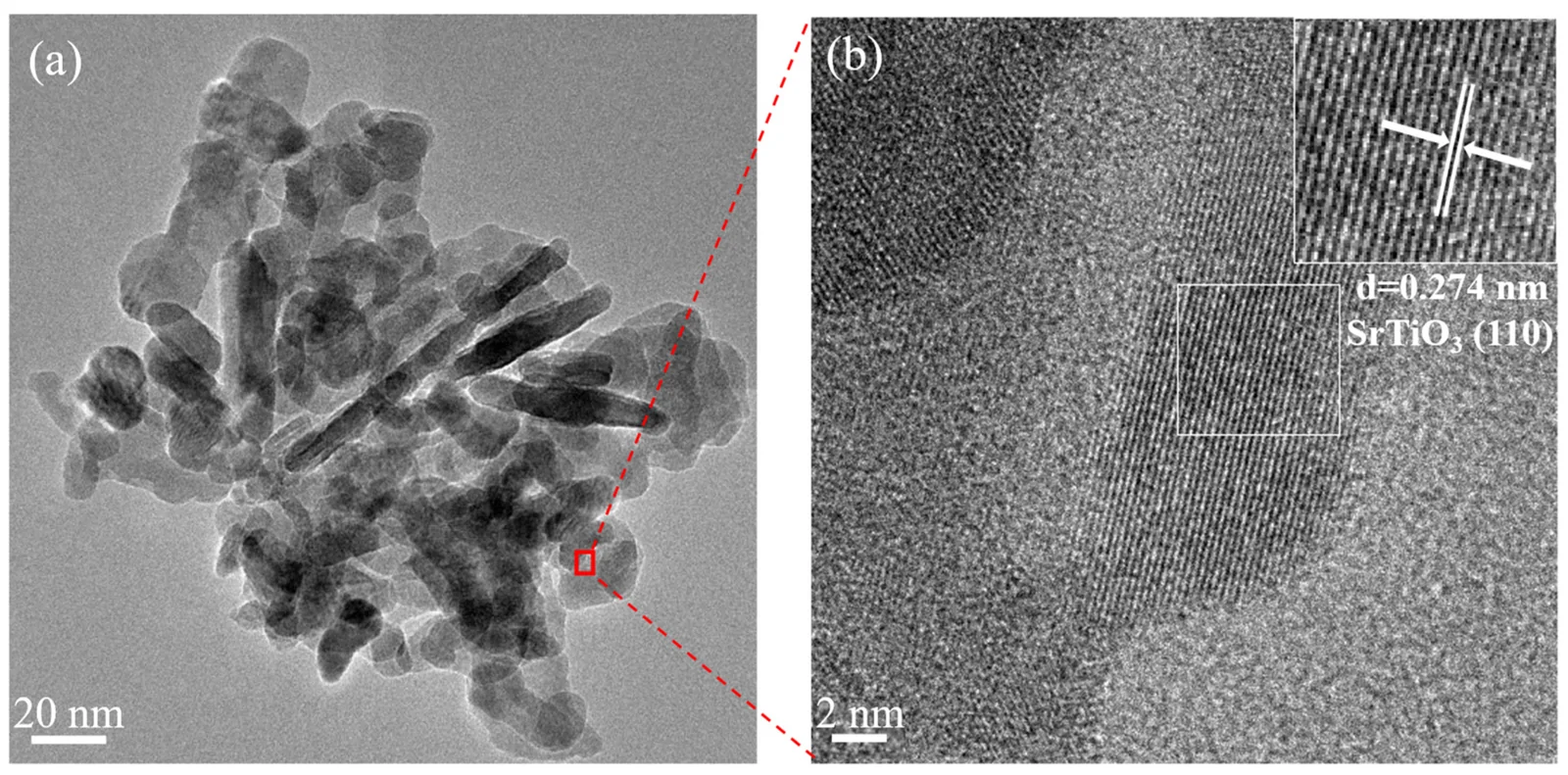
(**a**) TEM image and (**b**) HRTEM image of 0.5 wt%Ni/SrTiO_3_ sample.

TEM analysis of the 0.5 wt% Ni/SrTiO_3_ sample further confirms the short rod-like structure and close packing of the SrTiO_3_ particles. The relatively small particle size suggests the potential for more exposed active sites (Figure 2a). Systematic particle size statistical analysis was performed on the Ni-loaded samples. The particle size distribution (Figure S4) reveals that the dimensions fall predominantly within the 20–60 nm range, in good agreement with the SEM observations. The HRTEM image (Figure 2b) reveals clear lattice fringes with an interplanar spacing of 0.274 nm, which can be assigned to the (110) crystal plane of SrTiO_3_ [26]. It is worth noting that due to the close atomic numbers of Ni (28), Sr (38), and Ti (22), directly distinguishing highly dispersed Ni via contrast differences in conventional HRTEM imaging is extremely difficult [27]. Furthermore, within the observed field of view, no obvious Ni or NiO nanoparticles or clusters were detected.

**Figure 3:**
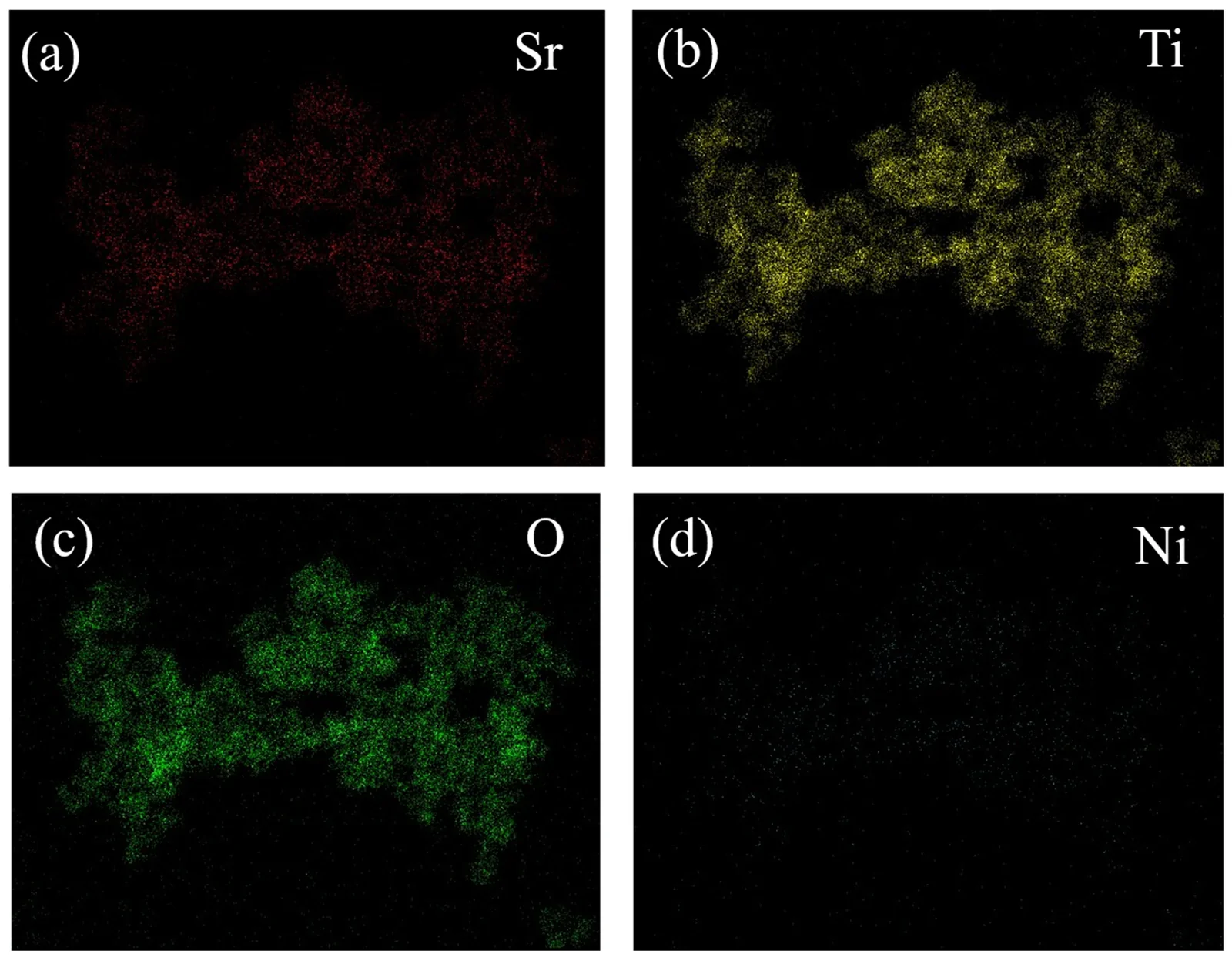
EDS elemental mapping of 0.5 wt%Ni/SrTiO_3_ sample: (**a**) Sr, (**b**) Ti, (**c**) O, and (**d**) Ni.

More importantly, as shown by the EDS elemental mapping in Figure 3d, Ni is homogeneously distributed throughout the specimen, which strongly supports the highly dispersed state of the Ni species on the SrTiO_3_ (Due to the low loading and highly dispersed state of Ni, its EDS signal appears weak in the uniformly displayed mapping. A clearer image after magnification and adjustment is provided in Figure S5). Taken together, the XRD results (absence of Ni/NiO crystalline peaks), XPS results (broadened and weak spectral features characteristic of Ni^2+^ species), and the aforementioned EDS mapping results collectively indicate that the nickel species exist in a highly dispersed state on the SrTiO_3_ support.

**Figure 4:**
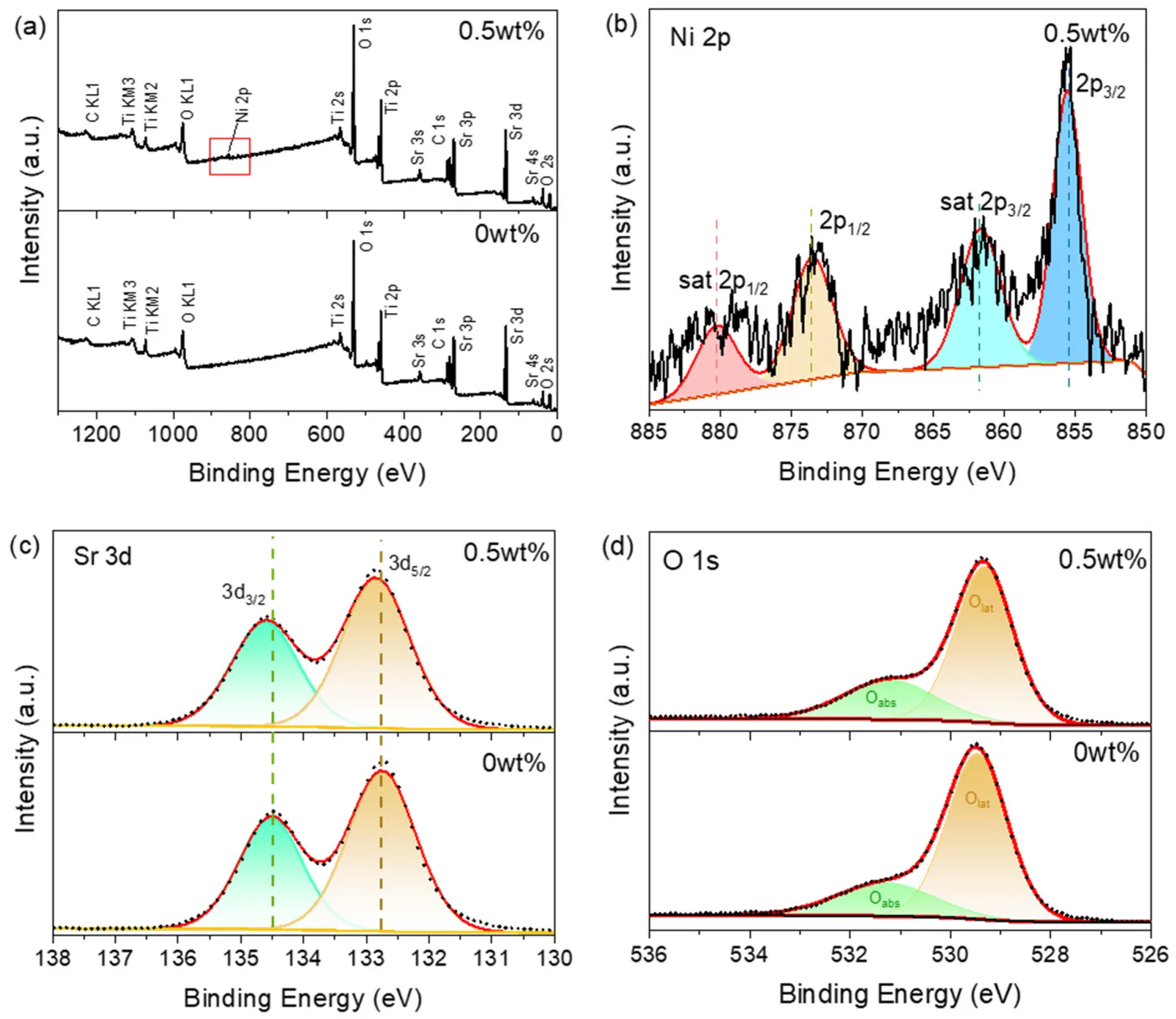
(**a**) XPS survey spectra of pure SrTiO_3_ and the 0.5 wt%Ni/SrTiO_3_ sample. (**b**) High-resolution Ni 2p spectrum of the 0.5 wt%Ni/SrTiO_3_ sample. (**c**,**d**) High-resolution Sr 3d and O 1s spectra of pure SrTiO_3_ and 0.5 wt%Ni/SrTiO_3_ samples, respectively.

The surface chemical composition of the samples was analysed using X-ray photoelectron spectroscopy (XPS). The obtained spectra were all charge corrected based on the C 1s peak at 284.80 eV. From the XPS survey scan shown in Figure 4a, characteristic signals of C, Sr, Ti and O elements were detected in both the pure SrTiO_3_ and 0.5 wt% Ni/SrTiO_3_ samples. The key distinction is the appearance of characteristic Ni 2p peaks around 855 eV in the Ni-loaded sample, indicating successful Ni loading. In the high-resolution Ni 2p spectrum (Figure 4b), peaks are observed at binding energies of 855.5 eV (2p3/2) and 874.0 eV (2p1/2), accompanied by satellite signals at 862.0 eV and 880.7 eV, which are attributable to Ni 2p3/2 and Ni 2p1/2, respectively. Compared with the sharp, strong peaks typically exhibited by NiO or metallic Ni nanoparticles, the broadened and weak intensity features of the Ni 2p signals in the samples are consistent with the common XPS characteristics of highly dispersed Ni species at low loading levels [25,28]. These results are mutually corroborated by other evidence, such as EDS mapping (uniform elemental distribution). Figure 4c shows Sr 3d XPS peaks at 132.6 eV and 134.5 eV, corresponding to Sr 3d5/2 and Sr 3d3/2 of Sr^2+^, respectively [29]. The high-resolution Ti 2p spectra (Figure S6) confirm the presence of only Ti^4+^ in both samples [29,30]. It is noteworthy that, compared with pure SrTiO_3_, small but noticeable changes in the binding energy position are observed in both the Sr 3d and Ti 2p peaks of the Ni-loaded samples. This variation may indicate the influence of an interaction between the Ni species and SrTiO_3_ support, more likely as Ni^2+^ as the samples was heated at high temperature in a vacuum, and subsequently exposed to atmosphere before loading into the XPS. Additionally, the vacuum calcination process indeed increases the Ni dispersion [31], as observed in Ni incorporated in Zeolite beta. Therefore, the sample preparation process described in this work supports the metal–support interactions, which can alter the electronic environment of the adjacent Sr and Ti atoms [32]. In the O 1s spectra (Figure 4d), peaks at 529.5 eV and 531.5 eV are assigned to lattice oxygen (Olat) and adsorbed oxygen species (Oads), respectively [33]. In the Ni-loaded samples, the relative content of adsorbed oxygen is 28.8%, which is higher than that in the pure SrTiO_3_ samples (24.7%), as shown in Figure S7. The rise in Oads may result from enhanced surface reactivity due to strong Ni–support interactions. 

**Figure 5:**
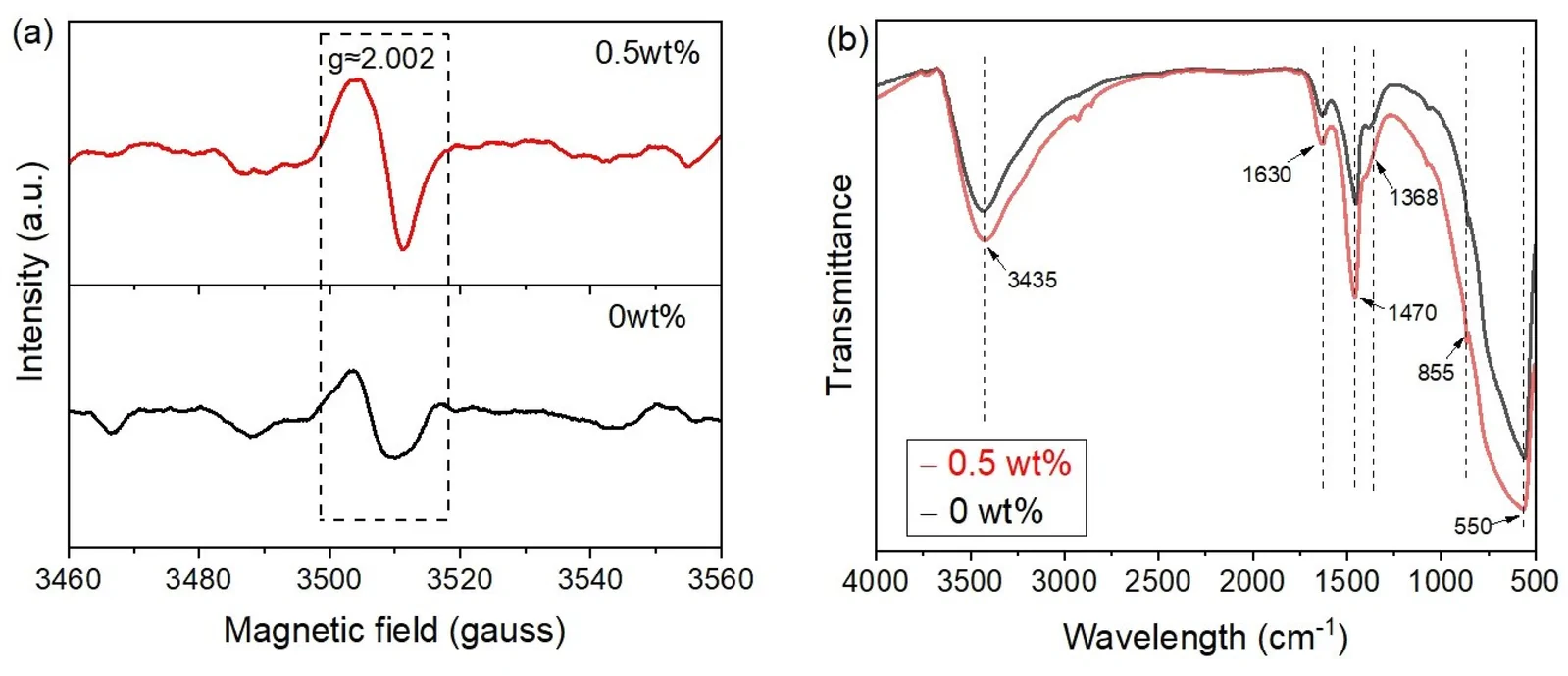
(**a**) EPR spectra and (**b**) FT-IR spectra of pure SrTiO_3_ and the 0.5 wt%Ni/SrTiO_3_ sample.

The 0.5 wt%Ni/SrTiO_3_ sample exhibited a higher concentration of V_o_ than pure SrTiO_3_. EPR measurements (Figure 5a) confirmed this, showing a stronger signal at g = 2.002 for the 0.5 wt%Ni/SrTiO_3_ sample, indicative of a higher V_O_ concentration. V_O_ on metal oxide supports often serve as anchoring sites for metal species [34], and the presence of such vacancies on pure SrTiO_3_ suggests that its surface can provide favourable sites for stabilizing nickel species. The increased oxygen vacancy concentration after nickel loading may be attributed to the interaction or substitution with Ti^4+^ through bridging oxygen between Ni^2+^ and the B site. The lower charge of Ni^2+^ likely induces the formation of oxygen vacancies (probably in the vicinity) for charge compensation [35], thereby generating more oxygen vacancies and consequently enhancing the photocatalytic activity [36]. The surface chemical states and structural bonding of the samples were further investigated by Fourier transform infrared (FT-IR) spectroscopy. To facilitate clear comparison, all measured spectra were normalized. As shown in Figure 5b, the spectral profiles of pure SrTiO_3_ and the 0.5 wt% Ni/SrTiO_3_ sample are similar, with the main differences lying in peak intensities and widths. The broad peak at 3435 cm^−1^ is attributed to the O-H stretching vibration of hydrogen-bonded water molecules, likely resulting from surface-adsorbed moisture upon exposure to air; surface hydroxyls can be a hydrophilic site that promotes water adsorption on the surface [37]. The peak at 1640 cm^−1^ corresponds to the frequency quoted for the bending mode of undissociated water molecules [38]. Peaks at 1470 cm^−1^ and 1368 cm^−1^ correspond to asymmetric and symmetric stretching of adsorbed CO_2_ [39]. These peaks are more intense in the 0.5 wt%Ni/SrTiO_3_ sample, indicating greater adsorption of H_2_O and CO_2_ and higher surface activity. Peaks around 855 cm^−1^ and 550 cm^−1^ are assigned to Sr-O and Ti-O bending vibrations and Sr-Ti-O stretching vibration, respectively [40]. The slight peak broadening observed in the 0.5 wt% Ni/SrTiO_3_ sample is likely due to enhanced interaction between the highly dispersed nickel species and the support. Furthermore, no new peaks attributable to Ni doping were detected, which further confirms that Ni is bonded to the support in a highly dispersed state rather than forming a separate independent phase [41].

### 3.2. Optical and Photocatalytic Properties

**Figure 6:**
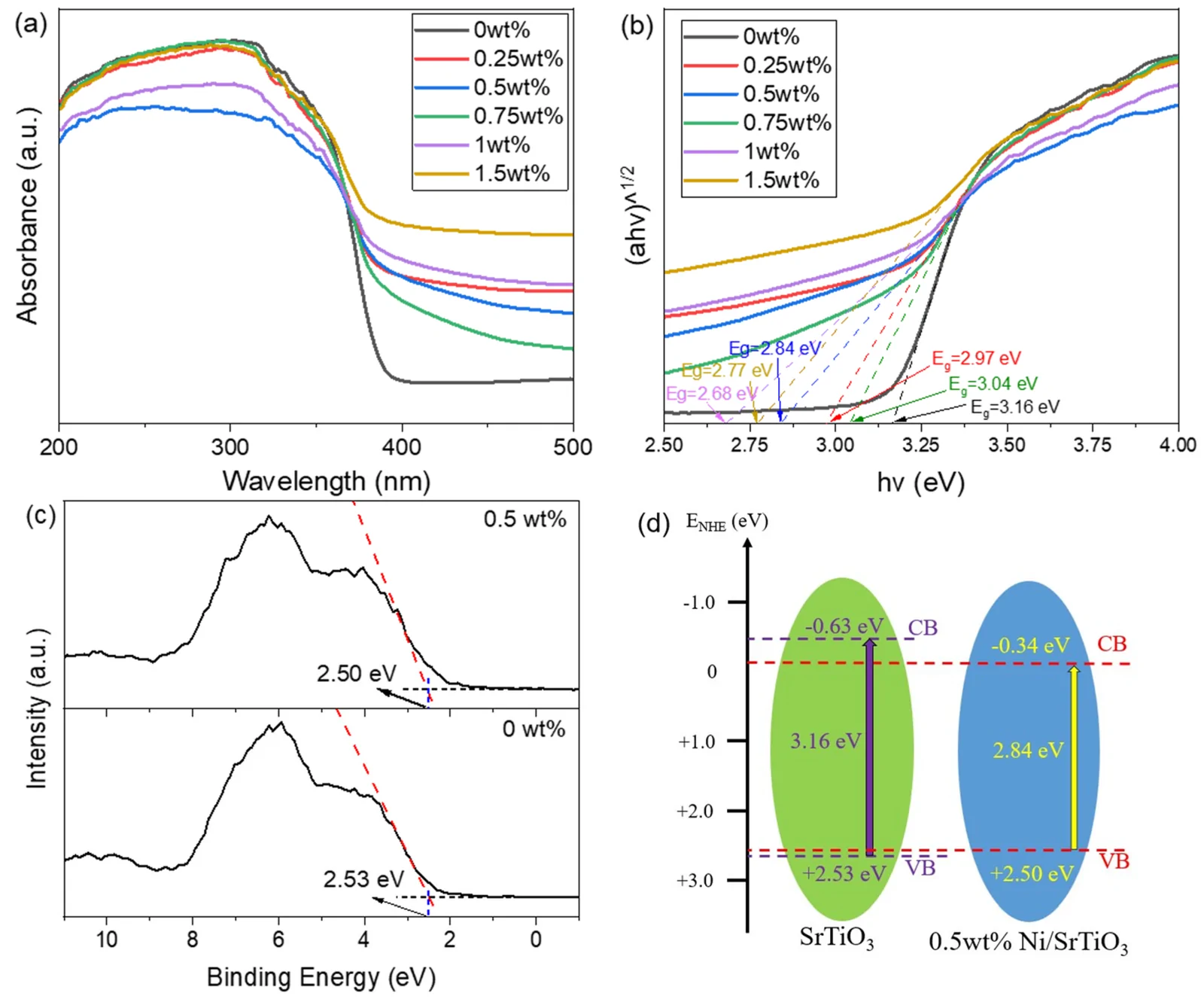
(**a**) UV-Vis absorption spectra; (**b**) derived optical band gaps; (**c**) VB-XPS valence band spectra and (**d**) energy band diagram of SrTiO_3_ and 0.5 wt% Ni/SrTiO_3_.

From the UV-Vis-NIR absorption spectra shown in Figure 6a, it can be observed that the light absorption of pure SrTiO_3_ is confined to the spectral region below 400 nm, demonstrating its inability to harvest visible or near-infrared photons. In contrast, Ni-loaded samples show stronger light harvesting in the visible spectrum (λ > 400 nm), suggesting an improved capacity to utilize visible light for generating more electron–hole pairs and influencing the formation and separation of photogenerated charge carriers [8]. The band gaps were calculated using the Kubelka–Munk formula: (αhν)^1/n^ = B(hν − E_*g*_), where α is the absorption coefficient, hν is the photon energy, B is a constant, and n depends on the nature of the optical transition [42]. As shown in Figure 6b, pure SrTiO_3_ has an optical band gap of 3.16 eV, close to its theoretical value (3.2 eV). After Ni loading, the band gap narrowed. This “apparent band gap narrowing” is primarily attributed to the introduction of nickel species, which leads to an increased concentration of oxygen vacancies, as evidenced by the EPR analysis (Figure 5a). Oxygen vacancies can act as shallow-level defect states, forming defect energy levels below the conduction band of SrTiO_3_ [43]. Valence-band X-ray photoelectron spectroscopy (VB XPS) was used to determine the valence band maximum (VBM) of the various samples (Figure 6c). The VBM values of pristine SrTiO_3_ and 0.5 wt% Ni/SrTiO_3_ are 2.53 eV and 2.50 eV (vs. NHE), respectively. Combining these with the corresponding band gap energies, the conduction band minima (CBMs) were calculated using the equation $E_{\mathrm{CB}}=E_{\mathrm{VB}}-E_{g}$, yielding values of −0.63 eV and −0.34 eV, respectively. Figure 6d presents a schematic diagram of the band energy alignment. Clearly, compared to pristine SrTiO_3_, the CBM of the 0.5 wt% Ni/SrTiO_3_ sample shifts downward by 0.32 eV toward the valence band [44]. Consequently, valence band electrons can be excited to these defect states by photons with energies lower than the intrinsic band gap, thereby enhancing visible-light absorption. Macroscopically, this manifests as an “apparent band gap narrowing” phenomenon, which ultimately improves the solar light utilization efficiency [45].

**Figure 7:**
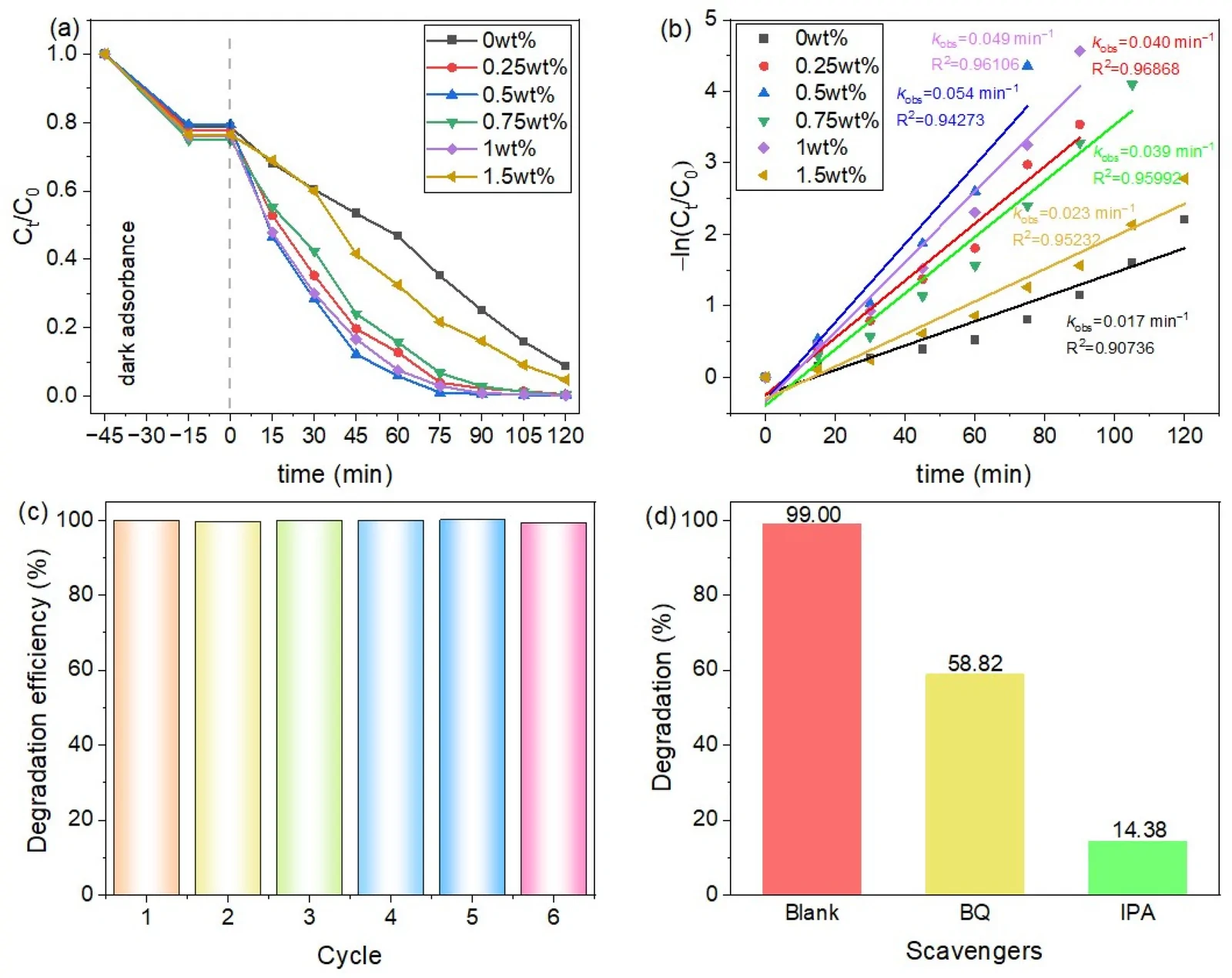
(**a**) Adsorption and photodegradation capabilities of the samples for MB. (**b**) Plots of -ln(C_t_/C_0_) versus irradiation time (*t*) for the samples. (**c**) Cycling performance of the 0.5 wt%Ni/SrTiO_3_ sample. (**d**) Trapping experiments using different scavengers for the 0.5 wt% Ni/SrTiO_3_ catalyst.

Figure 7a compares the photocatalytic performance of samples with 0–1.5 wt% Ni loadings. After 120 min of irradiation, neither pure SrTiO_3_ nor the 1.5 wt%Ni/SrTiO_3_ sample achieved complete degradation of methylene blue (MB). In contrast, samples with 0.25, 0.75, and 1 wt% Ni loadings removed nearly 100% of the MB within 90 min, while the 0.5 wt%Ni/SrTiO_3_ sample achieved full degradation in just 75 min, demonstrating significantly enhanced activity. The corresponding apparent photocatalytic rate constants (k) were calculated using the pseudo-first-order kinetics model [46]: −ln(C_t_/C_0_) = kt, where C_t_ and C_0_ represent the MB concentration at time t and the initial concentration, respectively. As illustrated in Figure 7b, the 0.5 wt% Ni sample exhibited the highest k value (0.05491 min^−1^), which is approximately 3.2 times that of the pure SrTiO_3_ sample (k = 0.01703 min^−1^), highlighting its superior photocatalytic efficiency. As demonstrated by the cycling tests in Figure 7c, the catalyst exhibited remarkable stability, retaining its catalytic performance after six repeated runs with no appreciable decline, which underscores its prospective application in photocatalytic wastewater treatment. Although direct quantification of Ni leaching was not performed in the present study, the strong metal–support interaction evidenced by the XPS and EPR analyses, together with the excellent cycling stability, suggests that the Ni species are firmly anchored on the SrTiO_3_ support and resistant to leaching during the photocatalytic process. To determine the dominant reactive species responsible for pollutant photodegradation, isopropanol (IPA) and benzoquinone (BQ) were employed as the quenching agent for •OH and •O_2_^−^, respectively, which are recognized as the primary oxidative species in the photocatalytic reaction [47,48]. As shown in Figure 7d, without any scavenger, the MB degradation efficiency reached 99.00%. Upon the addition of BQ, the degradation efficiency decreased to 58.82%, indicating that •O_2_^−^ plays an important role in the process. Notably, when IPA was added, the efficiency dropped significantly to 14.38%, suggesting that •OH is the predominant reactive species responsible for MB degradation [49]. As summarized in Table 1, the as-prepared 0.5 wt% Ni/SrTiO_3_ sample shows relatively high catalytic activity compared to other reported SrTiO_3_-based photocatalysts for organic pollutant degradation.

### 3.3. Photocatalytic Mechanism

**Figure 8:**
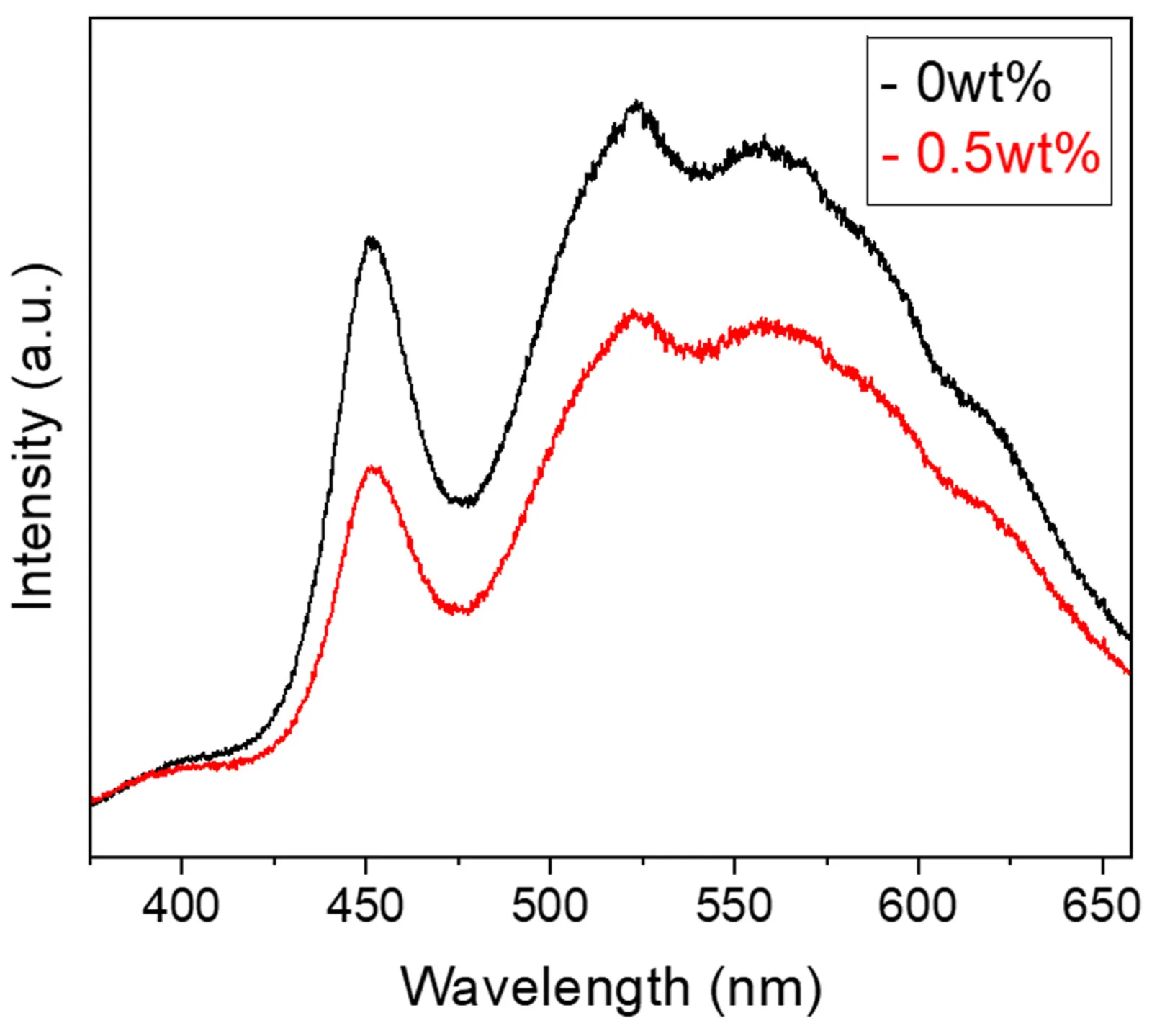
PL spectra of pure SrTiO_3_ and 0.5 wt%Ni/SrTiO_3_.

Photoluminescence (PL) spectra of SrTiO_3_ and 0.5 wt%Ni/SrTiO_3_ were collected to investigate charge carrier behaviour (Figure 8). The intense emission signal observed at around 450 nm can be assigned to band-to-band recombination, matching the intrinsic photoluminescence behaviour of SrTiO_3_ [57]. The broad emission between 500 and 600 nm originates from excitonic recombination related to surface oxygen vacancies and defects [58], and may also be influenced by particle aggregation. It is noteworthy that the 0.5 wt%Ni/SrTiO_3_ sample exhibits a much lower PL intensity than pure SrTiO_3_. Since weaker PL emission is generally indicative of higher charge separation efficiency [59], the marked suppression of photogenerated carrier recombination in the 0.5 wt%Ni/SrTiO_3_ sample allows more charge carriers to be available for photocatalytic reaction.

**Figure 9:**
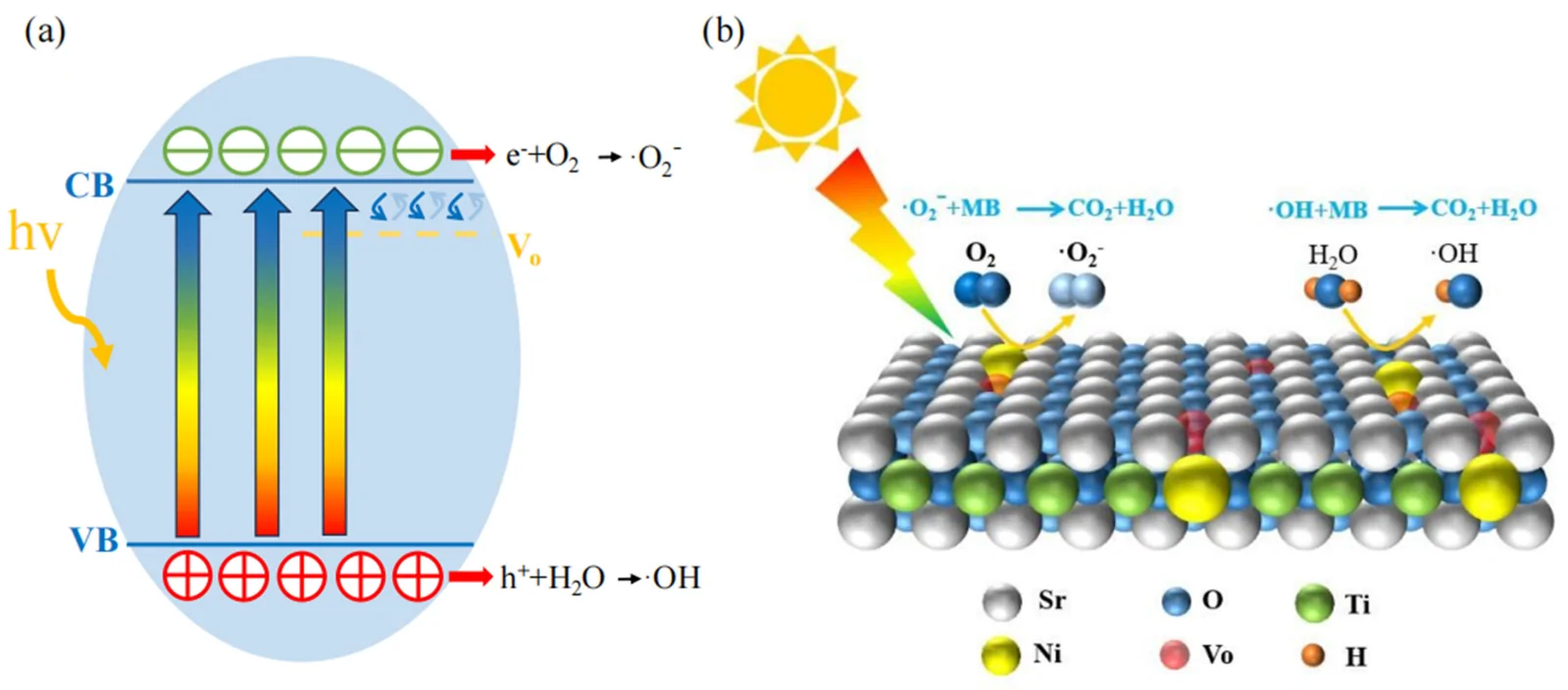
(**a**) Band structure schematic diagram of 0.5 wt%Ni/SrTiO_3_. (**b**) Photocatalytic mechanism of 0.5 wt%Ni/SrTiO_3_ for MB degradation.

Based on the above experimental results, the photocatalytic mechanism of highly dispersed Ni-modified SrTiO_3_ can be summarized from three key perspectives: optical absorption, charge dynamics, and surface reactivity [60]. Structurally, XRD, SEM, TEM, and EDS collectively confirm that the nickel species in 0.5 wt% Ni/SrTiO_3_ are anchored on the support surface in a highly dispersed, amorphous form. The electronic interactions revealed by XPS and the increased oxygen vacancy concentration confirmed by EPR together establish the structural foundation for a strong metal–support interaction. Under these circumstances, the highly dispersed nickel species, through their strong interaction with the support, induce the generation of abundant oxygen vacancies. These vacancies form defect energy levels within the bandgap of SrTiO_3_ (as shown in Figure 6), allowing electrons to be excited from the valence band to defect states by photons with an energy lower than the intrinsic bandgap. This results in an apparent narrowing of the bandgap (according to UV-Vis DRS results), thereby enhancing the photoabsorption efficiency in the visible spectrum [43,44,45]. Under simulated solar irradiation, photogenerated electrons transition from the valence band to the conduction band or localized defect levels. The photogenerated electrons can rapidly migrate to surface active sites through the strong interaction interface formed between the nickel species and the SrTiO_3_ support, where they participate in reactions such as the reduction of molecular oxygen to superoxide radicals (•O_2_^−^). Meanwhile, the photogenerated holes retained in the valence band are capable of oxidizing surface-adsorbed water or hydroxyl species to generate hydroxyl radicals (•OH). This process, on the one hand, shortens the electron transport pathway; on the other hand, through the synergistic effect of the respective reactions involving photogenerated electrons and holes, it significantly suppresses charge recombination [5]. In the photoluminescence (PL) spectra, the intensity of the 0.5 wt % Ni/SrTiO_3_ sample is markedly lower than that of pristine SrTiO_3_, confirming the above scenario. The electrons migrating to the surface participate in the reduction of molecular oxygen to generate superoxide radicals (•O_2_^−^), whereas the holes remaining in the valence band can oxidize surface-bound water or hydroxyl groups, resulting in the production of •OH. Trapping experiments clearly demonstrate that both species are the main active radicals responsible for the degradation of methylene blue, with •OH playing a particularly critical role. •O_2_^−^ and •OH jointly attack methylene blue molecules, gradually oxidizing and degrading them into small-molecule products such as CO_2_ and H_2_O [61]. The highly dispersed nickel species, which interact strongly with the support, optimize the distribution and accessibility of surface active sites [62], enhance the material’s ability to adsorb and activate H_2_O and O_2_ [63], and accelerate the photocatalytic reaction kinetics. These combined effects collectively contribute to the superior photocatalytic performance of the Ni/SrTiO_3_ material, as illustrated in Figure 9.

## 4. Conclusions

In summary, this study successfully synthesized a series of SrTiO_3_-based photocatalysts loaded with highly dispersed nickel species via a simple and controllable ammonia-assisted annealing method. The optimized 0.5 wt% Ni/SrTiO_3_ achieved complete degradation of methylene blue within 75 min under simulated sunlight, with an apparent reaction rate constant approximately 3.2 times higher than that of pure SrTiO_3_, along with good cycling stability. The characterization results reveal that the nickel species exist in a highly dispersed state and form strong interactions with the support, which significantly increases the surface oxygen vacancy concentration and introduces defect energy levels. These effects lead to a narrowing of the apparent band gap and enhanced visible-light absorption. Mechanistic studies indicate that the highly dispersed nickel species effectively promote the separation and transport of photogenerated charge carriers and, through enhanced surface reactivity, accelerate the generation of active species such as •OH and •O_2_^−^, thereby markedly improving the photocatalytic performance. This work provides new insights for designing efficient and stable SrTiO_3_-based composite photocatalysts.

## Figures and Tables

**Table 1:** Comparison of the photocatalytic performance of strontium titanate-based photocatalysts for organic pollutant degradation.

Material	Light Source	Time (min)	Pollutant	Degradation Rate (%)	Reference
Ni/SrTiO_3_ (Highly dispersed)	Xenon lamp	75	Methylene blue (10 ppm)	100%	This work
Ni/SrTiO_3_ (Nanoparticles)	UV lamp	330	Methylene blue (3.5 ppm)	99%	[50]
Bi/SrTiO_3_ (Doped)	Visible light	135	Acid Orange 7 (25ppm)	98%	[51]
Eu/SrTiO_3_ (Doped)	Xenon lamp	240	Rhodamine B (5ppm)	95%	[52]
Cu/SrTiO_3_ (Doped)	Visible light	120	Methyl Violet (10ppm)	99%	[53]
Rh/SrTiO_3_ (Doped)	Visible light	90	Phenol (10ppm)	100%	[54]
B/SrTiO_3_ (Doped)	Visible light	90	Phenol (10ppm)	73%	[55]
Ag/SrTiO_3_ (Doped)	Xenon lamp	120	Ciprofloxacin antibiotics 20ppm	84.5%	[56]

## Data Availability

The original contributions presented in this study are included in the article/Supplementary Material. Further inquiries can be directed to the corresponding author.

## Supplementary Materials

The following supporting information can be downloaded at: https://www.mdpi.com/article/10.3390/ma19183914/s1. Figure S1: Enlarged XRD patterns of pure SrTiO_3_ and 0.5 wt% Ni/SrTiO_3;_ Figure S2: SEM images of (a) SrTiO_3_ and (b) 0.5 wt% Ni/SrTiO_3;_ Figure S3: Statistical analysis of particle size (SEM); Figure S4: Statistical analysis of particle size (TEM); Figure S5: Magnified EDS mapping of Ni; Figure S6: High-resolution Ti 2p XPS spectra of the SrTiO_3_ and 0.5 wt% Ni/SrTiO_3_ samples; Figure S7: High-resolution O 1s spectra analysis of pure SrTiO_3_ and 0.5 wt% Ni/SrTiO_3_ samples; Figure S8: (a) XRD patterns and (b) MB adsorption and photocatalytic degradation capabilities of the samples with 0 wt%, 0.5 wt%, and 5 wt% Ni loadings; Figure S9: Photocatalytic degradation performance of 0%, 0.25%, 0.5%, 0.75%, 1%, and 1.5 wt% Ni/SrTiO_3_ samples; Figure S10: Photocatalytic degradation performance of 0%, 0.5%, and 5 wt% Ni/SrTiO_3_ samples; Table S1: Amounts of each reagents and annealing time used in the preparation of Ni/SrTiO_3_ samples using the impregnation-pyrolysis method.
