# Supplementary material for: Enhanced Photocatalysis over SrTiO3 Nanoparticles Modified with Highly Dispersed Nickel Species
Source: Materials (Basel). 2026 Sep 15;19(18):3914. doi: 10.3390/ma19183914 (PMC13608880; doi:10.3390/ma19183914)
Supplement: Supplementary file 1 [file materials-19-03914-s001.zip › materials-4501136-supplementary.pdf]

Supporting information for

Enhanced Photocatalysis over $SrTiO_3$ Nanoparticles Modified with Highly Dispersed Nickel Species

Yanlong Gao [1], Hongfei Wu [1], Senwei Wu [1], Xinhong Chu [2], Xiujian Zhao [3], Gopinathan Sankar [4] and Shouqin Tian [1,*]

[1] State Key Laboratory of Advanced Glass Materials, Wuhan University of Technology, Wuhan 430070, China
[2] College of Intelligent Manufacturing and Materials & Chemical Engineering, Yichun University, Yichun 336000, China
[3] State Key Laboratory of Silicate Materials for Architectures, Wuhan University of Technology, Wuhan 430070, China
[4] Department of Chemistry, University College London, London WC1H 0AJ, UK
* Correspondence: tiansq@whut.edu.cn

Academic Editor: Raphaël Schneider

Received: 30 July 2026
Revised: 6 September 2026
Accepted: 11 September 2026
Published: 15 September 2026

Copyright: © 2026 by the authors. Submitted for possible open access publication under the terms and conditions of the Creative Commons Attribution (CC BY) license.

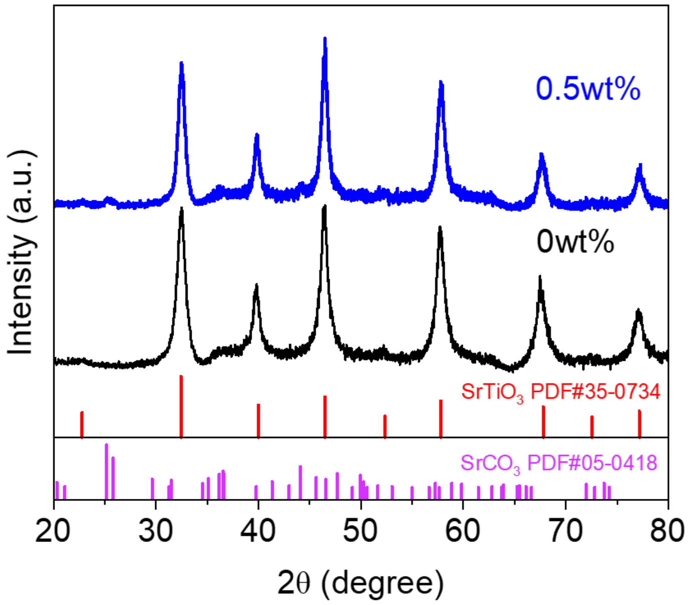

Figure S1 Enlarged XRD patterns of pure $SrTiO_3$ and 0.5wt% Ni/$SrTiO_3$.

The weak diffraction peaks observed at approximately 25° and 36° can be attributed to trace amounts of strontium carbonate ($SrCO_3$, PDF#05-0418), which originated from the reaction of residual ethylene glycol (from the hydrothermal synthesis) with strontium species on the $SrTiO_3$ surface during the subsequent annealing process.

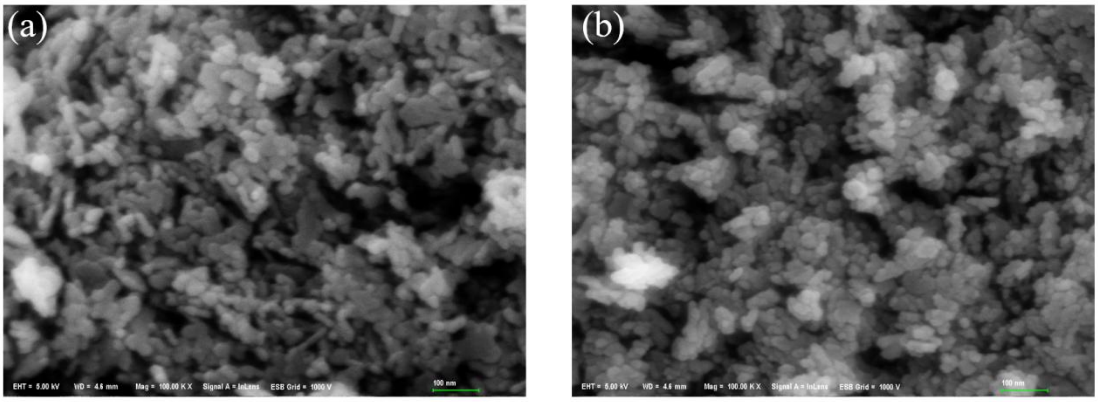

Figure S2. SEM images of (a) $SrTiO_3$ and (b) 0.5 wt% Ni/$SrTiO_3$.

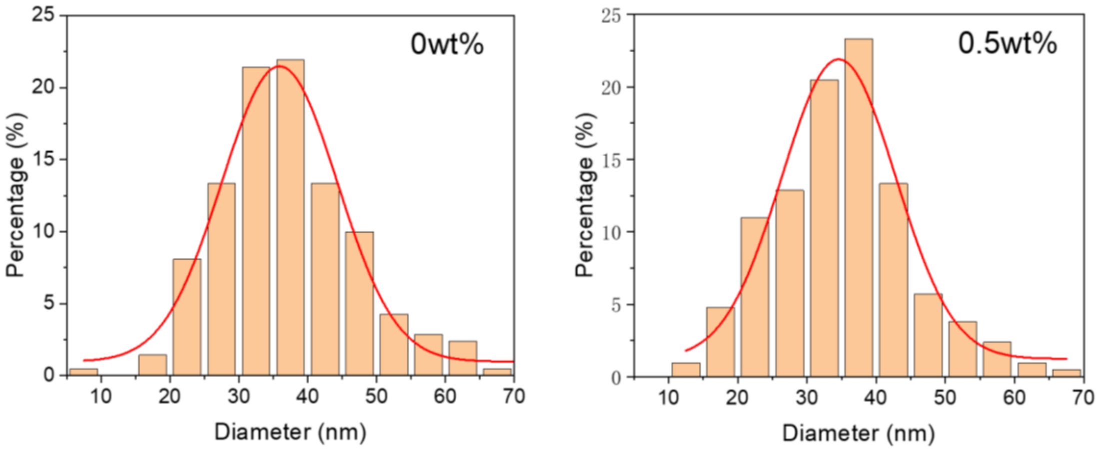

Figure S3 Statistical analysis of particle size (SEM).

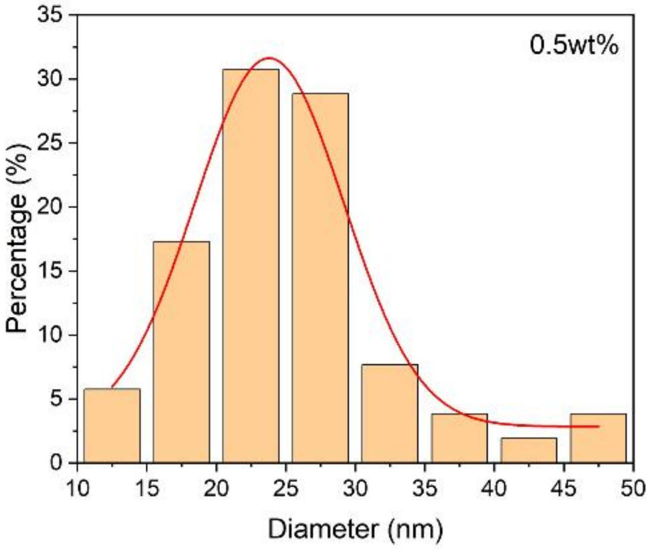

Figure S4. Statistical analysis of particle size (TEM).

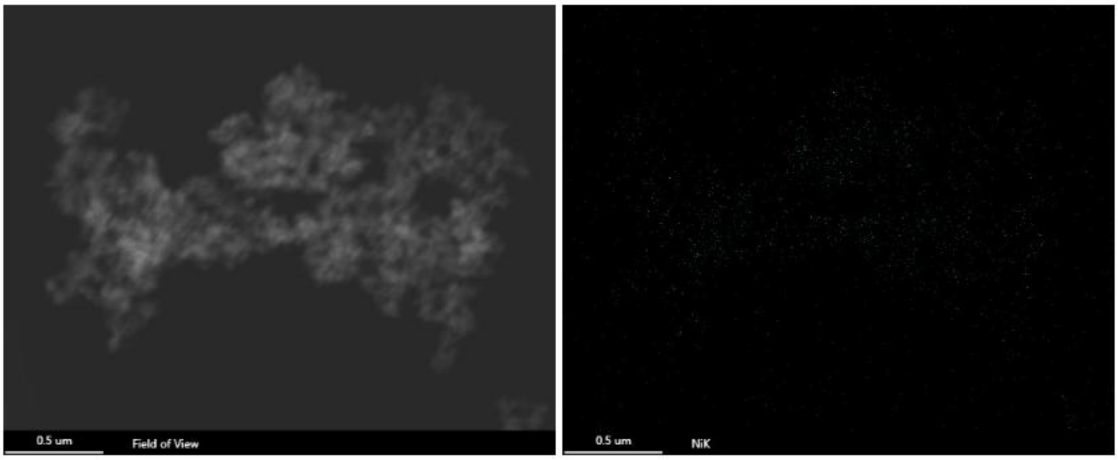

Figure S5 Magnified EDS mapping of Ni.

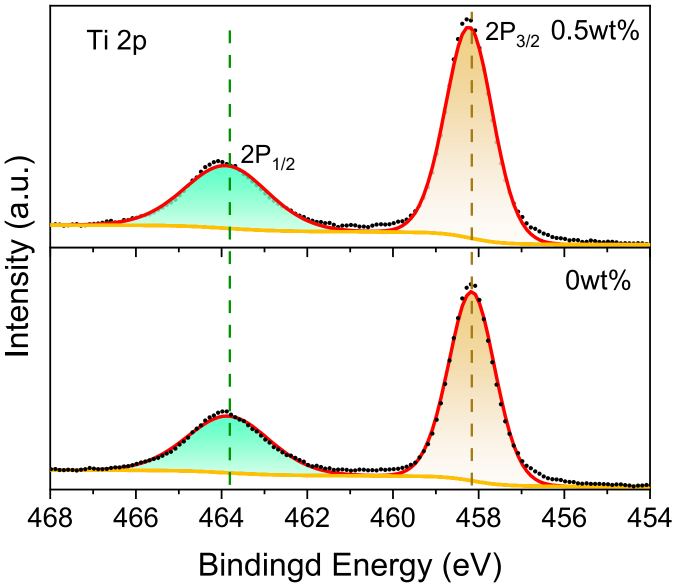

Figure S6 High-resolution Ti 2p XPS spectra of the $SrTiO_3$ and 0.5 wt% Ni/$SrTiO_3$ samples.

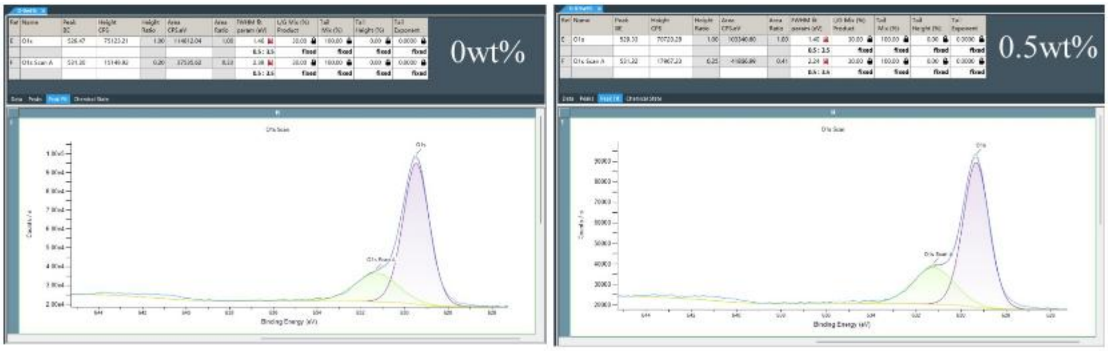

Figure S7 High-resolution O 1s spectra analysis of pure $SrTiO_3$ and 0.5 wt% Ni/$SrTiO_3$ samples.

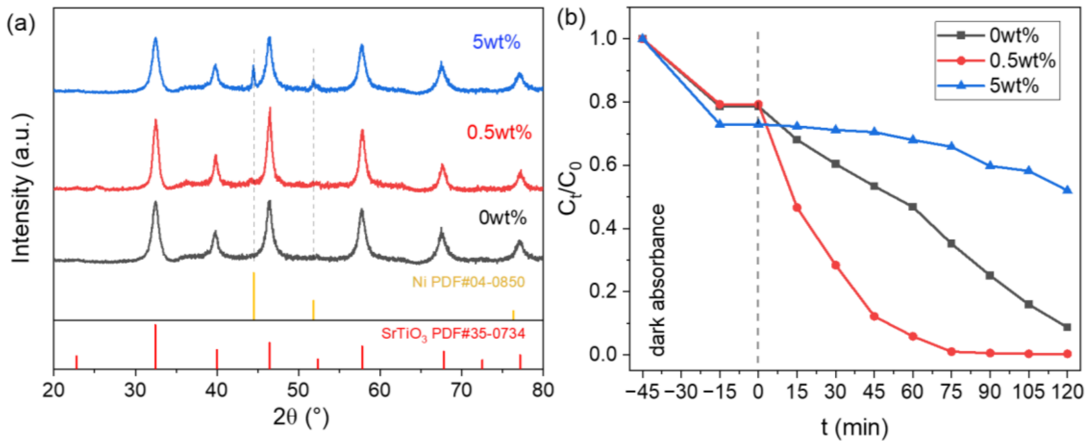

Figure S8 (a) XRD patterns and (b) MB adsorption and photocatalytic degradation capabilities of the samples with 0 wt%, 0.5 wt%, and 5 wt% Ni loadings.

To further substantiate the role of atomically dispersed Ni, a comparative experiment was conducted by increasing the Ni loading to 5 wt%. **Figure S8a** shows the XRD patterns of the samples with 0 wt%, 0.5 wt%, and 5 wt% Ni loadings. It can be clearly observed that when the loading increases to 5 wt%, distinct characteristic diffraction peaks of metallic Ni appear at 44.5° and 51.8°, indicating the formation of Ni crystals. Photocatalytic degradation experiments were performed on these samples, and the corresponding degradation rate constants are shown in **Figure S8b**. It is evident that the formation of metallic Ni does not enhance the performance of $SrTiO_3$; instead, it leads to a significant deterioration in photocatalytic performance. This result further confirms the crucial role played by atomically dispersed Ni in this study.

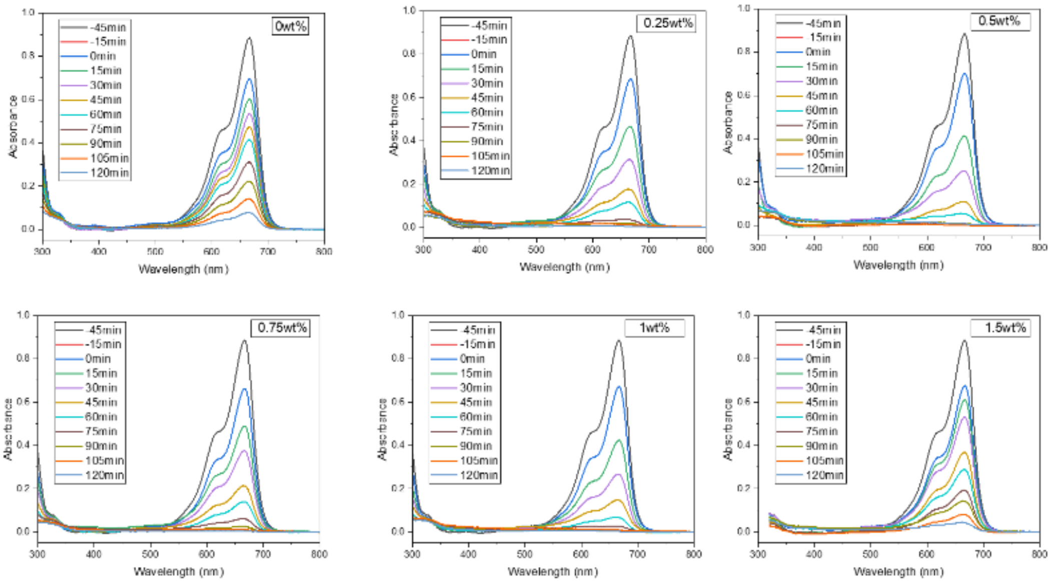

Figure S9 Photocatalytic degradation performance of 0%, 0.25%, 0.5%, 0.75%, 1%, and 1.5 wt% Ni/$SrTiO_3$ samples.

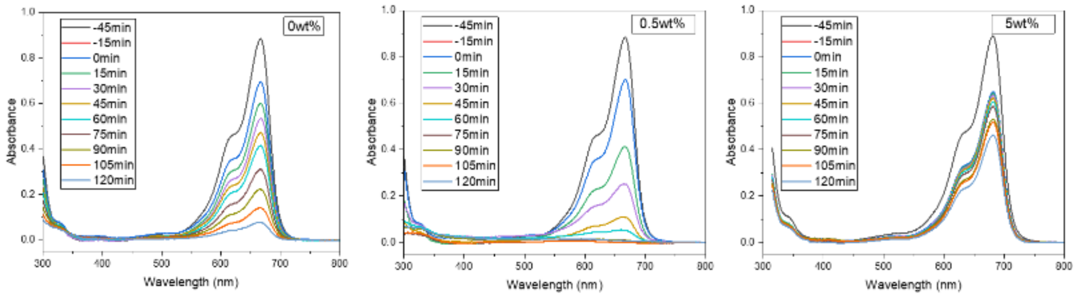

Figure S10 Photocatalytic degradation performance of 0%, 0.5%, and 5 wt% Ni/$SrTiO_3$ samples.

Table S1 Amounts of each reagents and annealing time used in the preparation of Ni/$SrTiO_3$ samples using the impregnation-pyrolysis method.

Sample	$Ni(NO_3)_2·6H_2O$	$SrTiO_3$	NH_4HCO_3	Annealing time/h
0.25wt%Ni/STO	6.2 mg	0.5 g	2 g	2
0.50wt%Ni/STO	12.5 mg	0.5 g	2 g	2
0.75wt%Ni/STO	18.6 mg	0.5 g	2 g	2

1.00wt%Ni/STO	24.8 mg	0.5 g	2 g	2
1.25wt%Ni/STO	37.2 mg	0.5 g	2 g	2
